# Colorectal carcinogenesis: Review of human and experimental animal studies

**DOI:** 10.4103/1477-3163.49014

**Published:** 2009-03-26

**Authors:** Takuji Tanaka

**Affiliations:** Department of Oncologic Pathology, Kanazawa Medical University, 1-1 Daigaku, Uchinada, Ishikawa 920-0293, Japan. E-mail: takutt@kanazawa-med.ac.jp

**Keywords:** Colorectal cancer, human and animal investigation, pre-neoplasia

## Abstract

This review gives a comprehensive overview of cancer development and links it to the current understanding of tumorigenesis and malignant progression in colorectal cancer. The focus is on human and murine colorectal carcinogenesis and the histogenesis of this malignant disorder. A summary of a model of colitis-associated colon tumorigenesis (an AOM/DSS model) will also be presented. The earliest phases of colorectal oncogenesis occur in the normal mucosa, with a disorder of cell replication. The large majority of colorectal malignancies develop from an adenomatous polyp (adenoma). These can be defined as well-demarcated masses of epithelial dysplasia, with uncontrolled crypt cell proliferation. When neoplastic cells pass through the muscularis mucosa and infiltrate the submucosa, they are malignant. Carcinomas usually originate from pre-existing adenomas, but this does not imply that all polyps undergo malignant changes and does not exclude *de novo* oncogenesis. Besides adenomas, there are other types of pre-neoplasia, which include hyperplastic polyps, serrated adenomas, flat adenomas and dysplasia that occurs in the inflamed colon in associated with inflammatory bowel disease. Colorectal neoplasms cover a wide range of pre-malignant and malignant lesions, many of which can easily be removed during endoscopy if they are small. Colorectal neoplasms and/or pre-neoplasms can be prevented by interfering with the various steps of oncogenesis, which begins with uncontrolled epithelial cell replication, continues with the formation of adenomas and eventually evolves into malignancy. The knowledge described herein will help to reduce and prevent this malignancy, which is one of the most frequent neoplasms in some Western and developed countries.

## Introduction

A neoplasm (or tumor) is a disease that is characterized by excessive and uncontrolled growth and spread of structurally and biologically abnormally differentiated cells that can originate from any tissues of the body. Benign neoplasms show a close morphological resemblance to their tissue of origin, grow in a slow expansive fashion and form circumscribed and (usually) encapsulated masses. They may stop growing and regress. Benign tumors do not infiltrate through local tissues and do not metastasize. They are rarely fatal. In contrast, malignant neoplasms, including cancer, resemble their parent tissues less closely and are composed of increasingly abnormal cells in terms of their cellular/structural form and function. Well-differentiated examples still retain recognizable features of their tissue of origin, but these characteristics are progressively lost in moderately and poorly differentiated malignancies; undifferentiated or anaplastic tumors are composed of cells that resemble no known normal tissue. Most malignant tumors grow rapidly, spread progressively through adjacent tissues and metastase to distant sites. Tumors are conventionally classified according to the anatomical site of the primary tumor and its microscopic appearance rather than by the cause. Cancer that is of epithelial origin is caused by both external and internal factors. These causal factors may act together or in sequence to initiate and/or promote cancer. In spite of knowing more than ever about the genetic and cellular events that can accelerate or inhibit cancer induction, cancer is still the number one health concern in the world, especially in Western and Westernized countries.

**Figure 1:**
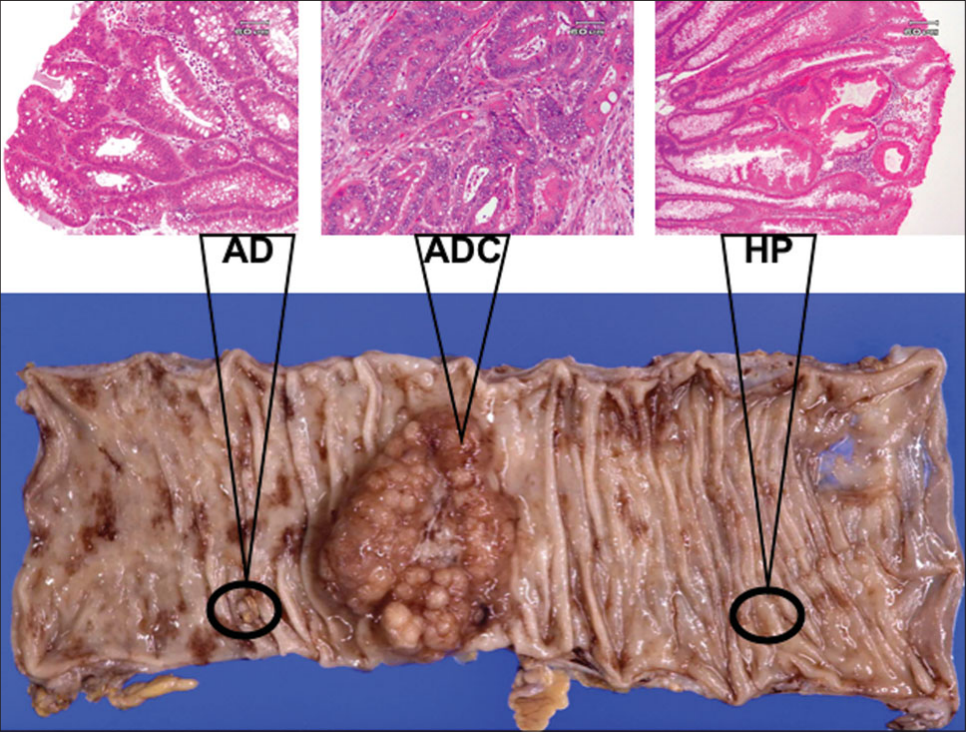
Colonic carcinoma, histopathologically diagnosed as moderately differentiated tubular adenocarcinoma (ADC) that developed in a 65-year-old Japanese male. Note an adenoma (AD) and a hyperplastic polyp (HP) surrounding the carcinoma

Cancer mortality rates in the developed countries have increased throughout the last century. It is already the leading cause of death in some Western and developed countries.[1,2] In Japan, the progressive introduction of Western dietary habits, especially increased fat intake and reduced carbohydrate and dietary fiber intake, has increased the incidence of colorectal cancer (CRC) [Figure 1] and related deaths.[3] Great advances have been made in the pharmacological-based treatment of malignant epithelial neoplasms (cancers). In addition, there is a marked increase in the understanding of cell and molecular mechanisms underlying the carcinogenic processes. However, therapy for advanced neoplastic disease remains limited. This may be due to the fact that advanced neoplasms, including CRC, contain a large number of genetic and molecular alterations that contribute to their neoplastic progression. In order to treat or control cancer, it is necessary to know how cancers occur and develop in the body. Of course, early detection of malignancies in different tissues is quite important for reducing the disease.[4]

This review will focus on CRC among a variety of cancers because this epithelial malignancy is one of the major cancers in developed countries and summarize recent findings of colorectal oncogenesis from human and experimental animal investigations.

## Epidemiology

CRC is more prevalent in North America, Argentina, Australia, New Zealand and parts of Europe, Japan and Israel, and, for this reason, is commonly regarded as a Western lifestyle disease. However, although the incidence and mortality are higher in countries with a Western lifestyle, the global incidence is rising and the majority of the world's cases of CRC occur outside of countries in which traditional Western lifestyles are dominant. CRC is one of the most frequent cancers worldwide. In the United States, CRC is the second leading cause of cancer-related death in men and the third in women.[5] In Japan, the westernization of lifestyles, especially dietary habits, has progressed remarkably since 1950, and this change is presumably directly related to the increasing incidence of CRC. In comparison to 1975, the age-adjusted incidence rates for colon and rectal cancers were estimated to be 3.7- and 1.9-times higher among men and 2.9- and 1.3-times higher among women by 1995 or 2000, respectively, and then to plateau. Considering the progressive aging of the society in Japan, the numbers of incident cases of CRC among men and women have been predicted to increase 9.5- and 7.5-times by 2005 and 12.3- and 10.5-times by 2020, respectively, from the 1975 baseline.[6]

## Colon (large intestine): Function, anatomy and morphology

The intestinal tract consists of the small intestine (duodenum, jejunum and ileum) and the large intestine or colon. The colon is covered by the peritoneum except for the most distal part, the rectum. The length of the human colon is of the order of 100–150 cm. The principal functions of the large intestine are the recovery of water and salt from feces and the propulsion of increasingly solid feces to the rectum before defecation.

**Figure 2:**
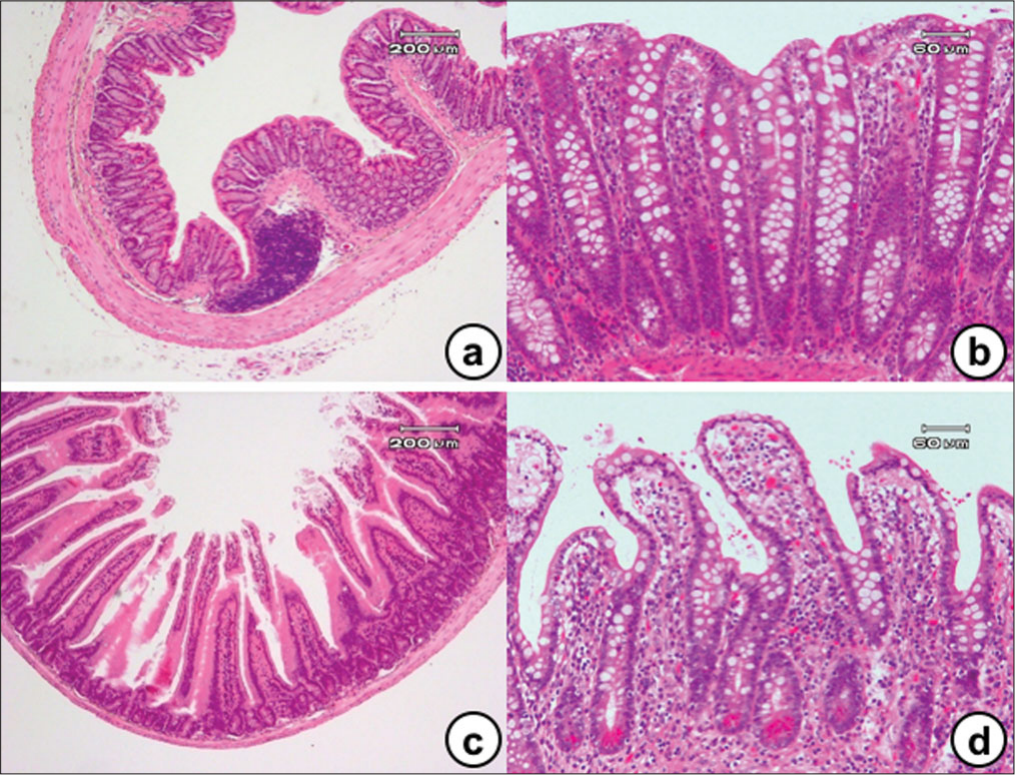
The histology findings of the colonic mucosa (a and b) and small intestine (c and d). While the villi are lined by a simple columnar epithelium that is continuous with that of the crypts in the small intestine, the colon lacks this structure (villi). The cell types in epithelium of the small intestine include: enterocytes, goblet cells, Paneth's cells, neuroendocrine cells, stem cells and intra-epithelial lymphocytes. The lamina propria extends between the crypts (a, b) and into the core of each villus (b) and contains a rich vascular and lymphatic network into which digestive products are absorbed. The muscularis mucosa lies immediately beneath the base of the crypts

As shown in Figure 2a and b, the colonic mucosa is folded in the non-distended state, but it does not exhibit distinct plicae circulares like those of the small intestine [Figure 2c and d]. Immediately above the anal valves, the mucosa forms longitudinal folds. The muscularis mucosae is a prominent feature of the large intestinal mucosa. Rhythmic contractions prevent clogging of the glands and enhance expulsion of mucus. The muscular wall is consequently thick and capable of powerful peristaltic activity. As in the rest of the gastrointestinal tract, the muscularis propria of the large intestine consists of inner circular and outer longitudinal layers but, except in the rectum, the longitudinal layer forms three separate longitudinal bands called teniae coll.

Consistent with its functions of water absorption and fecal lubrication, the mucosa consists of two types of cells: absorptive cells and mucus-secreting goblet cells. These are arranged in closely packed straight tubular glands or crypts, which extend to the muscularis mucosa [Figure 2a and b]. As feces pass along the large intestine and become progressively dehydrated, the mucus (producing approximately 3 L/day) becomes increasingly important for protecting the mucosa from trauma. The Alcian blue method stains goblet cell mucus, while the absorptive cells remain poorly stained. Goblet cells predominate in the base of the glands, whereas the luminal surface is almost entirely lined by columnar absorptive cells. Transverse sections through the upper pan of the large intestinal glands highlight the closely packed arrangement of the glands in the mucosa. The tall columnar absorptive cells have oval basal nuclei. In contrast, goblet cell nuclei are small and condensed. Stem cells at the base of the glands continually replace the epithelium. Intra-epithelial T lymphocytes T are easily seen. The lamina propria fills the space between the glands and contains numerous blood and lymphatic vessels into which water is absorbed by passive diffusion. The lamina propria also contains collagen as well as lymphocytes and plasma cells. These form part of the defense mechanisms against invading pathogens along with intra-epithelial lymphocytes and lymphoid aggregates, which are smaller than the Peyer's patches, found in the lamina propria and submucosa. The large intestine is inhabited by a variety of commensal bacteria, which further degrade food residues. Bacterial degradation is an important mechanism for the digestion of cellulose in ruminants. However, in humans, most cellulose is excreted. Small quantities of fat-soluble vitamins derived from bacterial activity are absorbed in the large intestine.

## Cell renewal (turnover)

**Figure 3:**
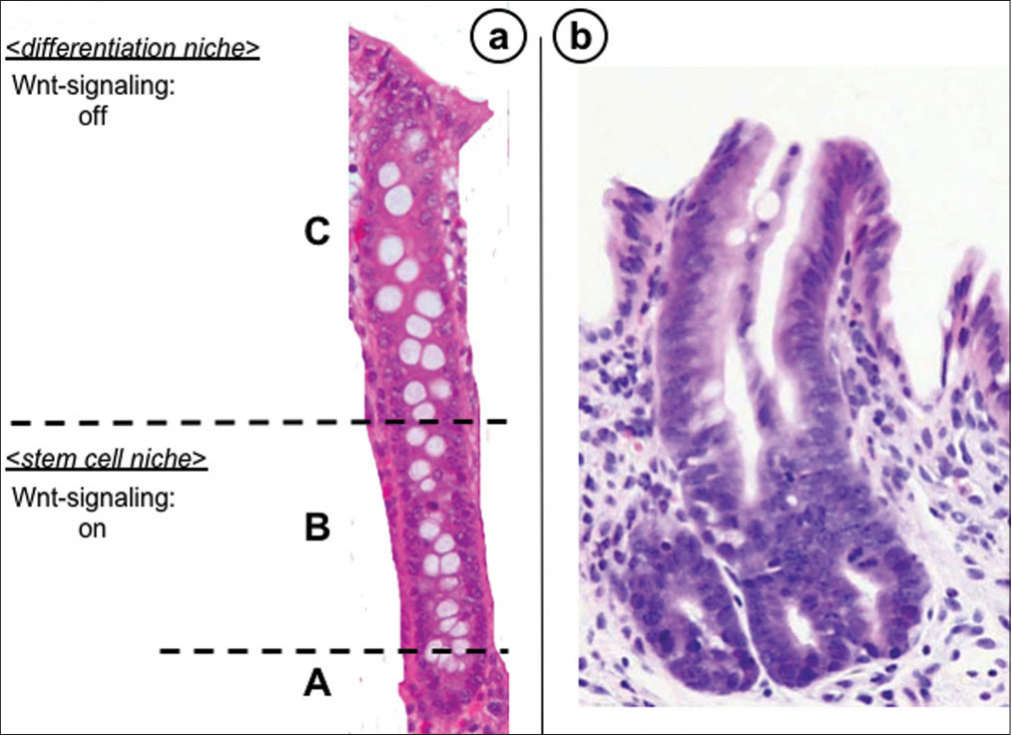
A high-power view of the colonic mucosa (a) and aberrant crypt fission in a familial adenomatous polyposis patient (b). (a) At the ‘A’ zone of the crypt, progenitor proliferating cells and stem cells are present. The ‘B’ zone contains transit-amplifying cells. On the ‘C’ zone of the crypt, there are mature and terminally differentiated cells. In the colonic epithelium, the proliferating crypt precursors and differentiated cells form a contiguous sheet of cells that is in perpetual upward motion. Stem cells reside near the bottom of the crypt and give rise to progenitor cells that are capable of differentiating toward all epithelial lineages. Proliferative progenitor cells arrest their cell cycle and differentiate when they reach near the top of the crypt. In this way, the large number of cells produced by the crypt compartment is compensated by apoptosis at the tip of the crypt in a process that requires about 2–3 days. (b) Aberrant crypt fission in a monocryptal dysplasia

The absorptive epithelium of the large intestine contains large numbers of cryptal cells. Differentiated cells (enterocytes, enteroendocrine cells and goblet cells) occupy the crypt [Figure 3a]. The remaining part of the crypts is made up of stem cells and the proliferating progenitor compartment [Figure 3a].[7] Stem cells reside near the bottom of the crypt and give rise to progenitor cells that are capable of differentiating toward all epithelial lineages [Figure 3a]. Stem cells self-renew to regenerate the epithelium after injury while progenitor cells arrest their cell cycle and differentiate when they reach the tip of the crypt.[8] The presence of these cells renders the colonic epithelium the most rapidly self-renewing tissue in adult mammals. Epithelial renewal occurs in the crypts through a coordinated series of events such as proliferation, differentiation and migration toward the large intestinal lumen.[9] In this way, the large number of cells produced by the crypt compartment is compensated by apoptosis at the tip of the crypt in a process that requires about 2–3 days. Proliferating crypt precursors and differentiated cryptal cells form a continuous sheet of cells in perpetual upward motion. Stem cells at the crypt bottom escape this flow.[10]

In abnormal states, the mucosa must also be able to repair mucosal integrity, which include the reversible ulcer-associated cell lineage, metaplasia (irreversible constitutive change of phenotype), dysplasia (abnormal differentiated phenotype) and neoplasia (abnormal cell number and abnormal differentiated phenotype). An abnormal pattern of cell replication has been detected in several clinical conditions associated with an increased risk for colorectal malignancies. These include patients with familial adenomatous polyposis (FAP),[11] individuals with adenomatous polyps of the large bowel (especially when large and multiple),[12] patients with long-standing inflammatory bowel disease (IBD),[13] normal individuals over 65 years of age[14] and patients who have previously undergone a polypectomy for colon polyp(s) or partial colonic resection for CRC.[15]

All the cells within the crypt are derived from the stem cells. One of the mitotic stem cells remains as a stem cell at the bottom of crypt and another cell is gradually pushed up to the luminal surface of the crypt as an epithelial cell. The cells that reached the uppermost part execute apoptosis and peel off without replicating or differentiating.[16,17] Therefore, any mutations in these cells have essentially no impact on the normal turnover of mucosa. The cells with damaged DNA (mutated genes) do not cause apoptosis and reach the uppermost part in the crypt and continue proliferating. This is a pre-cancerous change, aberrant crypt foci (ACF), and now being widely used as one of the biomarkers of colon carcinogenesis in chemopreventive experiments.[18–20]

## Stem cells and CRC

Over the past decade, the advances in the understanding of stem cell biology and the role of stem cells in diseases, such as CRC, have been remarkable. In particular, discoveries related to the control of stem cell proliferation and how dysregulation of proliferation leads to oncogenesis have been foremost. The WNT family of growth factors and events such as the regulation of the nuclear localization of β-catenin seem to be central to normal homeostasis in intestinal stem cells. Mutations in the components of these pathways lead to the development of CRC. A paradigm of abnormal stem cell biology is illustrated by patients with FAP, who have mutations in the adenomatous polyposis coli (*APC*) gene. The wild-type protein encoded by this gene is important for the prevention of mass β-catenin accumulation in the nucleus and the subsequent overtranscription of cell cycle proteins. These are the basic mechanisms behind stem cell regulation in the gut and their role in the natural history of tumor progression.

In FAP, which is associated with germline mutations in *APC*, the earliest visible change before the development of small, dysplastic adenomas is an upward shift in the proliferative compartment.[21] Mathematical models propose an increase in the number of intestinal stem cells as the origin of the observed hyperproliferation.[22,23] Using non-oncogenic mitochondrial DNA mutations as markers, it was elegantly shown that mutant clones initially expand by crypt fission.[24] Wnt signaling enhances the expression of proliferation-associated genes, such as *c-MYC* and *CyclinD1*, and, together with Notch signaling, regulates the switch of intestinal stem cells to transit-amplifying crypt progenitor cells. This supports the idea that *APC* mutations affect the regulation of intestinal stem cells. Furthermore, aberrant, asymmetric and increased crypt fission is detectable in FAP[25] [Figure 3b]. This is also associated with an increase of intestinal stem cells and leads to the proposal that *APC* mutations lead to a shift from an asymmetrical to a symmetrical division of intestinal stem cells.[26] Other models predict increased stem cell survival after *APC* mutation, which can be explained by enhanced expression of the Wnt target gene *survivin*, also considered to be a putative stem cell marker.[23]

**Figure 4:**
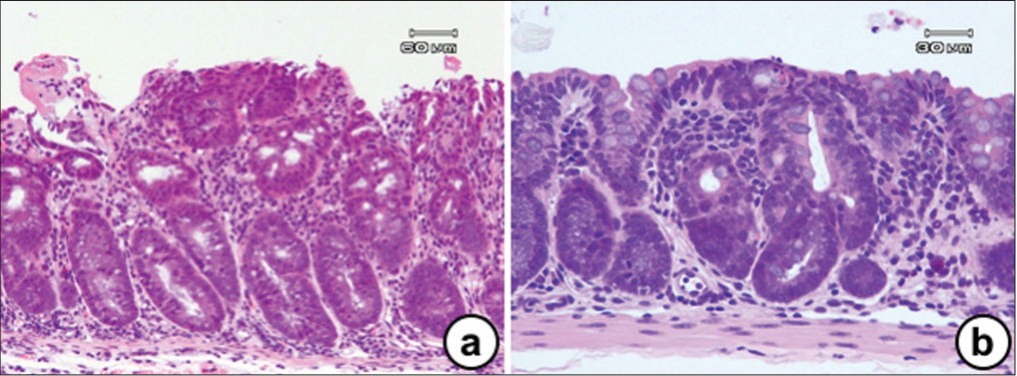
The histopathology findings for the ‘top-down’ (a) and ‘bottom-up’ (b) models. Atypical dysplastic cells are present in the upper (a) and lower (b) parts of the crypts

There are two models for the development of adenomas. In sporadic colonic adenomas, the initiating event seems to be loss of heterozygosity (LOH) at the *APC* gene, followed by a second hit in the *APC* gene. In one model, the ‘top-down’ model [Figure 4a], mutant cells appear in the intra-cryptal zone between crypt openings.[27] Only as the clone expands does it penetrate down into the crypt. In early non-FAP adenomas, dysplastic cells are found only at the orifices on the lumen surface of the crypts. In microdissected specimens, LOH for *APC* is found in the upper portions of the crypts in half the samples. Only the superficial cells show intense staining for β-catenin as evidence of a dysfunctional *APC* gene. Therefore, whereas the stem cell that is the likely oncogenic precursor must have originated in the base or depths of the crypt, the polyp originates in the top of the crypt or in the space between the crypts. There are two possibilities with the ‘top-down’ model. There may be the establishment of a new source of stem cells in the intra-cryptal zone. A terminally differentiated cell, thus, may be mutated to a fully competent dividing cell. On the other hand, a cell derived from a mutated stem cell may migrate to that area and with growth potential as a ‘second hit’ in the intra-cryptal zone, expand from this location. More likely, a stem cell with a mutational defect in growth control proliferates in the normal course of events and cells pushed up to the intra-cryptal area still retain this mutation, as do all cells between the stem cell at the base of the crypt all the way up to the intra-cryptal area and beyond. Perhaps more intuitive, the ‘bottom-up’ model [Figure 4b] is based on the crypt stem cell.[25] Commonly in FAP, but less so in non-FAP, the unicryptal adenoma with the dysplastic adenoma involving the entire crypt predominates. In FAP, non-cryptal lesions are clonal, as determined in an XO/XY individual. Sporadic and FAP adenoma appears to start as unicryptal adenomas and grows initially by fission of the crypt. Later, in sporadic adenomas, there is growth down into adjacent crypts. In OAT ^+/−^ individuals with FAP, there are increased rates of stem cell mutation and clustering of mutated crypts consistent with the ‘bottom-up’ model.[28]

## Colonic field cancerization

The initial mutation predisposing the tissue to CRC is likely to be disseminated over a wide distance. This would be analogous to the field effect in the head and neck and urinary tract cancers,[29] where the instance of second primaries in the adjacent normal tissues is extremely high. Whereas in the case of the field effects in CRC, head and neck cancer and urothelial cancer, the effect may be due to common exposure to environmental carcinogens (tobacco, alcohol, nitrates), there are considerable data now to suggest that the effect is due to proliferation of a common precursor or stem cell. Genetic changes characteristic of the first primary cancer extend into the normal mucosa adjacent to the cancer.[30] In head and neck cancer, these patches could extend 3–10 cm or perhaps farther from the edges of the primary cancer. This finding by itself has major implications for approaches to ‘localized’ cancer because in spite of a normal appearance (hence apparently adequate surgical margins) they may still contain the genetic or molecular alterations of the primary cancer . In an analysis of second primary cancers by microsatellite and markers such as *p53*, the second primary shows changes both identical to and different from the primary. A few cases show an identical base pair change in *p53*, which could not have happened by chance, strongly suggesting a clonal origin for the two separate primaries.[31] If it was due to carcinogen exposure alone, different specific base changes should have been found. Instead, both the first and second primaries must have arisen from the same cell, with subsequent accumulated additional genetic changes accounting for the dissimilarities between them.

Some experimental data show evidence of a field cancerization for CRC. CRCs contain a K-*ras* mutation in exon 12 in about 40–50% of the cases. In the normal mucosa, the same mutation is found in about 20% of the cases.[32] The field effect in CRC is similar to that in other cancers with pre-malignant lesions, in that spontaneous cancers usually arise in polyps and the incidence of recurrent or multiple polyps is very high. This would imply that the unique events causing a polyp (LOH in *APC*) have actually been diffusely spread from perhaps a single cell rather than by chance in multiple cells in the same field. Recently, field cancerization in human ulcerative colitis (UC)-associated tumors has been demonstrated by a mutation burden analysis.[33]

The field effect in head and neck cancer makes it a premier model for cancer prevention because reversal of the genetic changes in the apparently normal mucosa would effectively prevent progression to new malignancy. The likelihood of a similar field effect in CRC would have the same implications. Surrounding a primary tumor, or over a wide range of normal mucosa before the acquisition of the ultimate induction effect, areas of actually abnormal mucosa exist, with the potential for reversal of the defect. Whereas polyp removal can be a preventive measure, the recurrence of polyps is common, presumably due to the field effect in CRC. Reversal of those changes and repopulation by normal stem and crypt precursor cells would reverse the carcinogenic potential. Therefore, a second target for CRC prevention would be the reestablishment of effective DNA repair and relief of the microsatellite instability phenotype.

## Precursor or pre-malignant lesions of CRC

**Figure 5:**
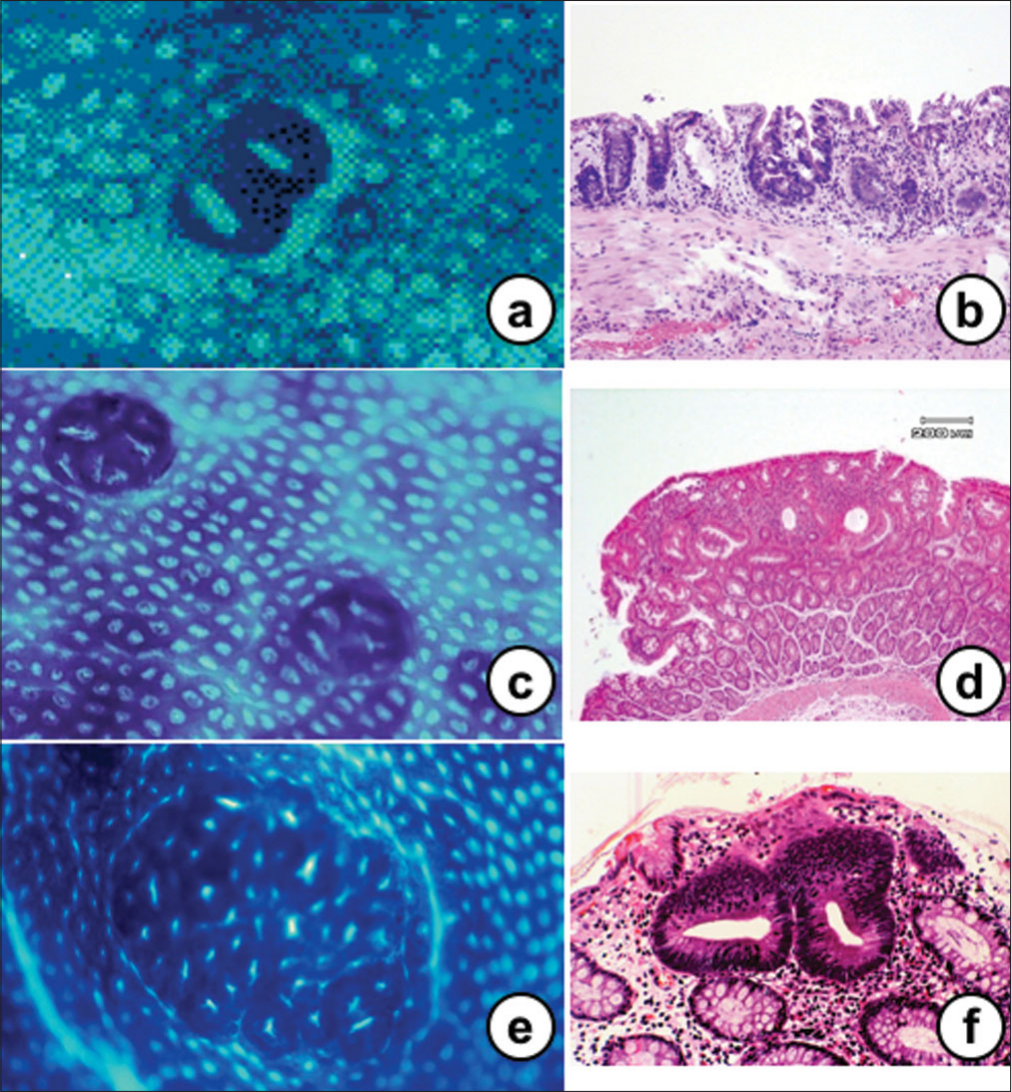
The histopathology findings of aberrant crypt foci (ACF) from mouse (a and b), rat (c and d) and human (e and f) colon. Methylene blue staining is capable of identifying ACF (a, c and e). These histopathology findings show dyplastic (b and f) and hyperplastic (b) characteristics. (e and f) Familial adenomatous polyposis patient

**Figure 6:**
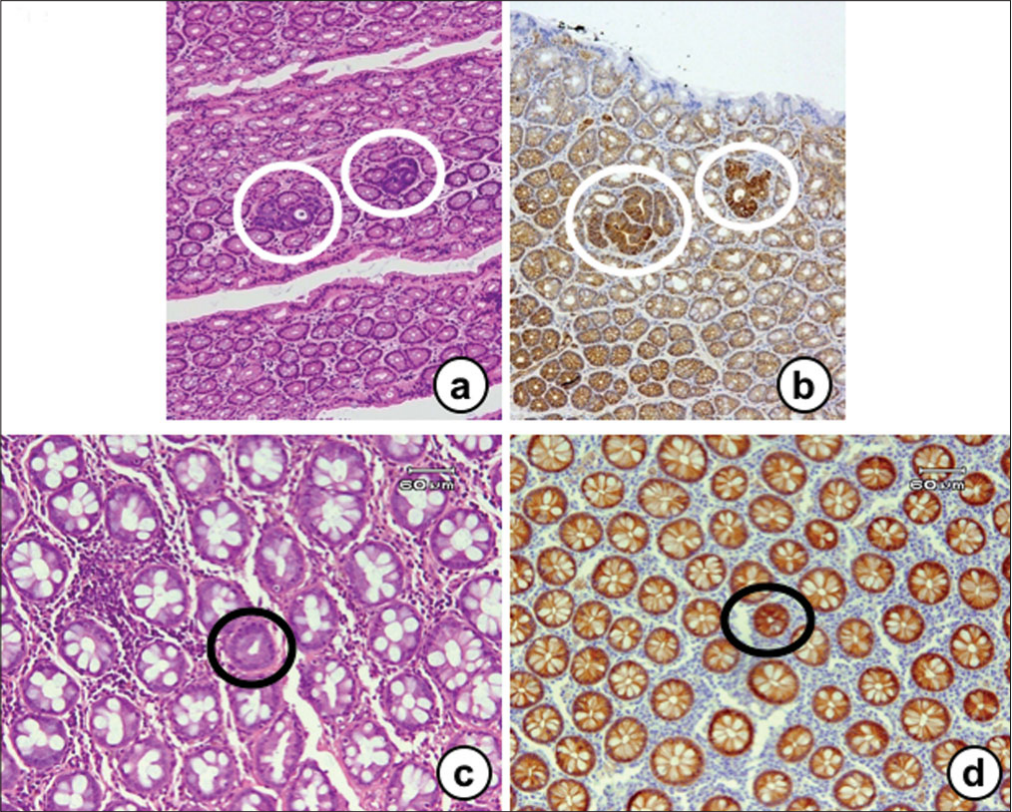
The histopathology and β-catenin-immunohistochemistry findings of BCAC from mouse (a and b) and human (c and d) colon specimens. BCAC (circled) can be detected on hematoxylin and eosin stain-stained sections (a and c) based on the intensive basophilic nuclei of atypical cells and a few goblet cells in the lesion. β-catenin-immunohistochemistry shows intensive reactivity in the nuclei and/or cytoplasms of atypical cells that form BCAC (b and d)

**Figure 7:**
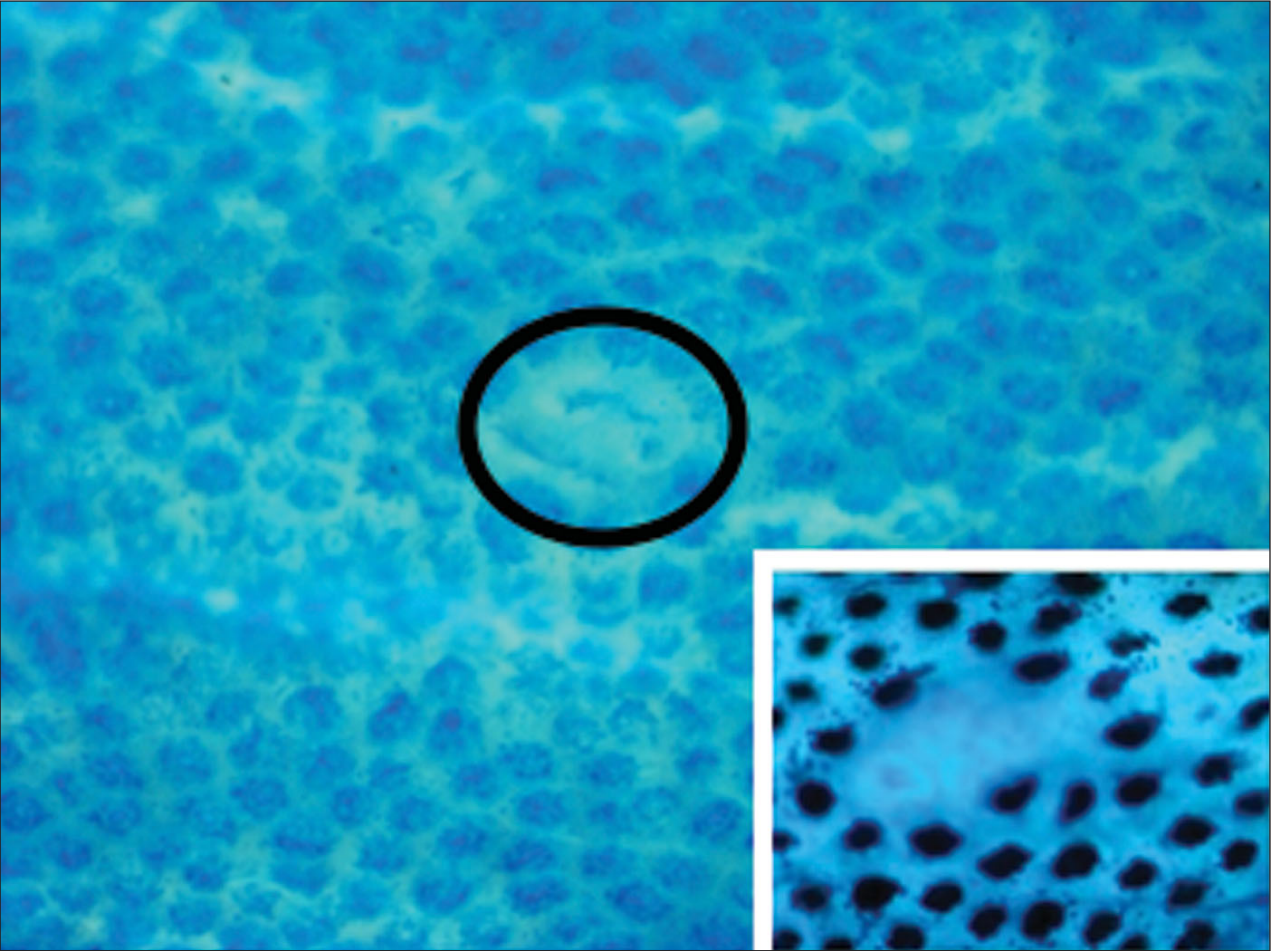
A MDF (circled) found in the human colon with colorectal cancer. MDF is identified as the lesion with negative staining with high-iron diamine Alcian blue. An ‘insert’ is a high-power view

Surrogate endpoint markers provide opportunities to understand cancer development and also to evaluate the efficacy of intervention. Based on epidemiologic, therapeutic, pathophysiological, clinical and cost benefit, adenomas are considered to be surrogate endpoints in CRC because removal of adenomatous polyps (adenomas) has been shown to reduce the risk of development of CRC. However, intervention using adenomas as the endpoint may not be fruitful because colonic adenomatous polyps often take several years to develop and become adenocarcinomas. Much interest is currently directed toward research in the use of surrogate endpoint biomarkers that are altered early in colonic carcinogenesis, before polyp (adenoma) formation, to predict the clinical effectiveness of chemopreventive agents or drugs because it takes 10–20 years for a normal cryptal cell to undergo molecular changes and to be clinically detected as a neoplasm. Rather, ACF [Figure 5] can be used as the endpoint in CRC development because aberrant crypts are postulated to be the earliest identifiable potential precursors of CRC in rodents and humans. The analysis of ACF may facilitate the study of the early pathological and molecular changes that precede the development of an adenoma to CRC.[34,35] Therefore, ACF may eventually evolve into polyps and, subsequently, CRC in the case of the ‘adenoma–carcinoma’ sequence. Hence, it provides a simple and economical tool for preliminary screening of potential chemopreventive agents and it allows a quantitative assessment of the mechanisms of colon carcinogenesis. β-catenin-accumulated crypts [Figure 6][36] and mucin-depleted foci [Figure 7][37,38] are also early lesions that develop into CRC[39], but the detection of these lesions from human specimens [Figures 6 and 7] is not easy by routine laboratory techniques. ACF and MDF are microscopically visible in the unsectioned colon stained with methylene blue and high-iron diamine Alcian blue, respectively.

## ACF and BCAC

ACF [Figure 5] are early appearing lesions recognized on the colonic lumen surface in rats treated with colon-specific carcinogens, azoxymethane (AOM), 1,2-dimethylhydrazine (DMH) and 2-amino-1-methyl-6-phenylimidazo [4,5-*b*] pyridine (PhIP).[20,40–43] These ACF are characterized by crypts with (1) altered luminal openings, (2) thickened epithelia and (3) being larger than the adjacent normal crypts.[44] The numbers of ACF increase with time after exposure to the carcinogen, but there is only weak evidence correlating tumor development with the expression of ACF. In rats or mice with a predisposition to develop CRC, histological sections (horizontal cross-sections) reveal dysplastic crypts with excessive β-catenin accumulation, called BCAC [Figure 6a]. These have disrupted cell morphology and increase with time after carcinogen treatment. BCAC do not have the appearance of ACF and are usually not recognizable on the mucosal surface.[36] BCAC have greater dysplasia than ACF and are associated with Paneth's cells, as are CRCs, but not normal colonic epithelium.[45] However, it is not clear whether BCAC represents a subgroup of ACF or can be clearly delineated as a separate entity. These foci are disseminated over several crypts, possibly by crypt fission or by cell migration. Clearly, not all these cells are malignant because the number of CRCs is less than the number of foci. There are many cells in each microadenoma or ACF/BCAC. If another mutation conferring carcinogenic potential occurs presumably it would be in a single cell and arise in the ‘field’ of abnormal cells (in particular, the i3-catenin-accumulating cells). The potential size of that ‘field’ is not known.

## Adenoma (adenomatous polyp)

The term ‘polyp’ should not be used by itself for histological diagnosis. ‘Polyp’ is a clinical description of any circumscribed mass of cells that project above the surface of the surrounding normal mucosa. Colorectal polyps can be defined as well-demarcated, circumscribed lumps of epithelial dysplasia with uncontrolled crypt cell division. Single transformed cells may generate subclones through new mutations, with the possibility of an enhanced growth rate. Most adenomas remain benign and asymptomatic lesions, which can be discovered by chance during lower endoscopy. However, a small fraction of these lesions may evolve into malignancy and there is evidence indicating that a large majority (if not all) of colorectal carcinomas develop from adenomatous polyps.[46]

**Figure 8:**
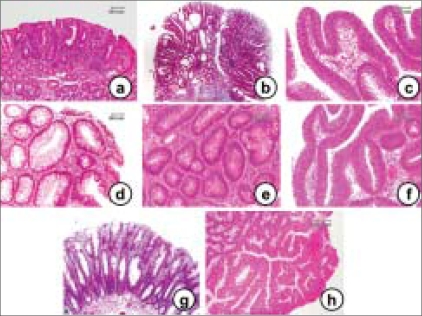
Various histological types (a–c) of human colonic adenomas with different degrees of dysplasia (d–f) and the histopathology of a hyperplastic polyp (g) and serrated adenoma (h). (a) Tubular adenoma, (b) tubule-villous adenoma, (c) villous adenoma, (d) tubular adenoma with mild-grade dysplasia, (e) tubular adenoma with moderate-grade dysplasia and (f) tubulevillous adenoma with severe-grade dysplasia. (g) The crypts of a hyperplastic polyp are elongated and exhibit cystic dilatation. The epithelium is composed of well-differentiated cells without any atypical features and shows a slightly serrated (saw-tooth) structure. The lesions are macroscopically as small as 0.5 cm in diameter and are always multiple. (h) Serrated adenoma possesses the same serrated architecture of hyperplastic polyps but shows dysplasia of the epithelium. The lesions are larger in size than hyperplastic polyps and are considered pre-malignant in nature, like other adenomas

Adenomas are well-demarcated lumps of epithelial tumor cells, which can be classified into the following three major histological types: tubular, villous and tubulo-villous adenomas [Figure 8a–c]. These three types, however, are not well differentiated from each other because they are only different manifestations of a spectrum of abnormal tissue architecture. An adenoma is pedunculated when it possesses a stalk. Sessile adenomas rise above the background mucosa without any stalk.

Adenomas, by definition, show different grades of dysplasia and should be classified taking into account the part with the most advanced grade. The word dysplasia is used to describe structural and cytological alterations in the epithelium that predispose an organ to cancer development. These abnormalities show varying degree of severity, which can be graded into ‘mild,’ ‘moderate’ and ‘severe’ atypia [Figure 8d–f]. It should be stressed that the terminology used for grading varies not only with the organ or tissue under investigation but also with the personal preference and attitude of the investigator. Notwithstanding these limitations, severe dysplasia in an adenoma is considered a selective marker for the increased risk of cancer and this is particularly true for lesions greater than 1.0 cm in diameter and with a marked villous component.[46] The definition of different grades of epithelial dysplasia in adenomas is one of the most convincing pieces of evidence of the ‘adenoma–carcinoma’ sequence. It is likely that most (if not all) colorectal carcinomas evolve through stages of increasingly severe epithelial dysplasia before becoming invasive lesions. Many molecular abnormalities have been reported in colorectal adenomas, which include mutations of oncogenes (such as k-*ras*), inactivation of tumor suppressor genes (such as *APC*, *p53* and *DCC*) and of mutator genes (repairing DNA mismatch), such as *MLH1* and *MSH2*, and disturbances of DNA methylation and microsatellite instability. Many of these genetic events may target the transition from normal mucosa to small adenomas and from these to large adenomas and infiltrating carcinoma.[47]

**Figure 9:**
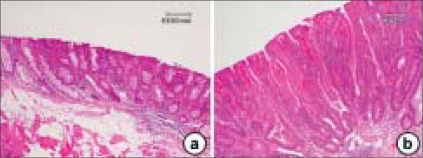
Histopathology findings of a flat adenoma (a) and a flat adenocarcinoma (b). These lesions are almost flat and are not detected by pre-operative endoscopic examination

A flat adenoma [Figure 9a] of the large bowel was initially described by Muto *et al*,[48] as slight elevations of the mucosa with a reddish surface that was dome-shaped and rather flat. Histopathologically, the polyp is tubular, villous or tubule-villous adenoma. Flat adenomas are difficult to detect in routine lower endoscopy because their shape may change with the degree of air insufflation. Nonetheless, recognition of these lesions is important because the malignant potential [Figure 9b] of flat adenomas seems to be considerably higher than that of common sessile or pedunculated polyps of the same size.[49] In addition, this provides further biological evidence favoring the ‘adenoma–carcinoma’ sequence. There are two primary macroscopic variants of flat adenomas, one that is completely flat[48] and the other that shows a central area of depression (depressed adenoma).[50] Depressed adenocarcinomas have also been reported.[51]

## Other lesions

Besides adenomatous polyps, other types of similar lesions deserve some comment either for their frequency [Figure 8g] or for their potential malignant evolution (flat adenomas) or for their peculiar histological features (hamartomas and juvenile polyps). Hyperplastic polyps are rounded and usually sessile lesions, a few millimeters in diameter, showing elongated crypts with a tendency to cystic dilatation. The epithelium is characterized by a single layer of cells, with no crowding of nuclei or signs of dysplasia. *In vitro* cell kinetic investigations together with electron-microscopy studies suggest that the elongation and subsequent infolding of the epithelium is attributed to an expanded but normally located proliferative zone in the crypt with a tendency to cystic dilatation. These lesions are most common in the rectum and sigmoid colon and become more frequent with increasing age. They cannot be distinguished from adenomatous polyps microscopically or by endoscopy.

It is commonly believed that hyperplastic polyps, especially those located in the rectum and sigmoid colon, are absolutely benign lesions that lack any malignant potential. However, there are various reports in the literature in which hyperplastic polyposis that is more often distributed in the various tracts of the large bowel is associated with the development of malignant tumors.[52] It is likely that, in these cases, adenomatous changes co-exist with hyperplastic features. As an alternative explanation, at least some hyperplastic polyps might carry an intrinsic potential for dysplasia and cancer.[53] Polyps combining features of both hyperplastic and adenomatous lesions are defined as ‘mixed polyps’ and may result from the engulfment of pre-existing hyperplastic polyps by spreading adenoma, stimulation of mucosal hyperplasia at the advancing edge of an adenoma or the development of an adenoma within a hyperplastic polyp. Each component in these lesions is histologically distinct from the other.

Longacre and Fenoglio-Preiser[54] described the morphological features of colorectal hyperplastic-adenomatous polyps that are occasionally observed throughout the colorectum. The general architecture of these polyps is similar to that of hyperplastic polyps, but the cytological features are different because the surface mitotic activity, nuclear pseudostratification and nuclear/cytoplasmic ratio are greater than in classic hyperplastic lesions. Moreover, signs of dysplasia are observed in almost 40% of the cases. Because one common feature of these polyps is the presence of a serrated glandular pattern, they termed the polyps as ‘serrated adenoma’ [Figure 8h] in order to stress their neoplastic nature and their possible malignant evolution. In keeping with these observations, recent studies show various biomolecular alterations in the hyperplastic polyps, including k-*ras* and *p53* mutations, LOH and microsatellite instability.[53]

## Polyps and dysplasia in IBD

**Figure 10:**
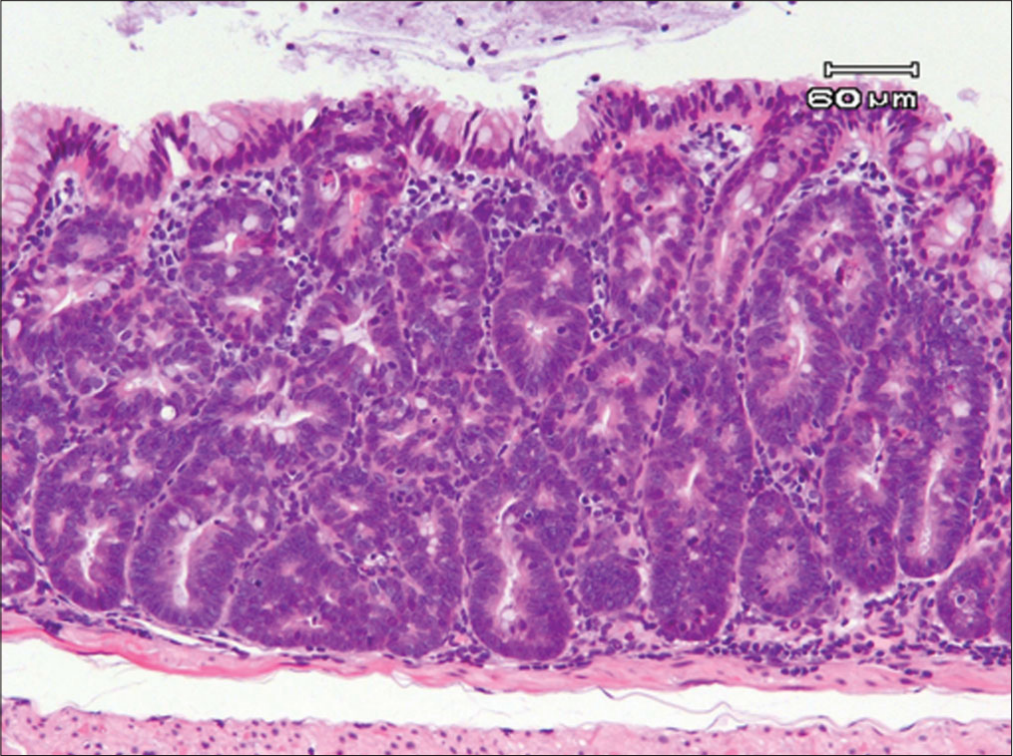
The histopathology findings of a dysplasia-associated lesion or mass developed in the colon of a Japanese ulcerative colitis patient. Note that the lesion is composed of many dysplastic glands

Patients with UC and Crohn's colitis of Crohn's disease (CD) are at increased risk for colorectal malignancies, the risk increasing with the duration of the disease and the extent of colorectal involvement.[55] The morphological basis of tumor occurrence is the development of dysplastic changes in the flat mucosa or in polypoid lesions. In IBD, elevated, sessile and reddish nodules, which are known as pseudopolyps or inflammatory polyps, are often seen in the otherwise flat mucosa. These lesions are typically small and multiple and largely composed of granulation tissue, mixed with inflamed and hyperemic mucosa. Dysplasia may grow as a flat lesion [Figure 10] or as a dysplasia-associated lesion or mass (DALM). DALM has to be distinguished from sporadic adenoma that is not the consequence of a chronic inflammation but an age-related coincidental finding. The presence of dysplasia in the flat mucosa adjacent to the DALM is the criterion for distinguishing DALM from adenoma.[56] The clinical distinction between DALM and sporadic adenoma that may occur in patients with IBD is extremely important. Indeed, the former lesion arises as the result of a chronic inflammatory stimulus in a patient with UC or Crohn's colitis. Recently, several molecular alterations have been detected in long-standing UC. These include oncogene mutations, inactivation of tumor suppressor genes, LOH and chromosomal and microsatellite instability.[57]

## Human colon carcinogenesis

In the USA, average-risk patients account for approximately 75% of CRC and include persons older than 50 years with no other known risk factor, moderate-risk patients account for 15–20% of CRC and include those with a positive family history of colorectal adenomatous polyps or cancer and high-risk patients account for 5–15% of CRC and include those with FAP, hereditary non-polyposis colorectal cancer (HNPCC) or long-standing IBD.[58,59] Therefore, the majority of CRC are non-hereditary and sporadic, which makes early detection important. There is good evidence demonstrating reduced morbidity and mortality associated with early detection of invasive lesions and precursor adenomatous polyps. However, most CRC in the world is diagnosed at an advanced stage. Therefore, most attention has focused on screening for targets for cancer chemoprevention to reduce the number of CRC patients.

Most sporadic CRCs (‘adenoma–carcinoma’ sequence) develop through multi-step and multi-genetic alterations. Recent advances in cancer pathogenesis help us in untangling the valid enzymatic and molecular biomarkers that are involved in colon tumorigenesis. The identification/discovery of these biomarkers ranges from exposure assessment, risk assessment and management to clinical trials. This is helpful for us to develop and discover novel therapeutic interventions, preventive strategies and chemopreventive agents. Along with these, there is also a need to develop and validate molecular biomarkers reflective of exposure and risk from etiological factors.[60–64] There are high-risk people who are most susceptible to chronic disease, including cancer. Hence, methods that identify such high-risk individuals will greatly facilitate the implementation of a spectrum of targeted prevention techniques directed to reducing individual risk. Currently developed technologies, -omics (genomics and proteomics) and miRNAs, are presently guiding the development of agents that are also able to target the biomarkers. Specific molecular processes have been targeted for therapeutic intervention, including growth factor receptors, proliferation signaling, cell cycling, apoptosis, angiogenesis, the immune system, etc. Most cancers are characterized by alternations in certain signaling pathways and identification of the aberrant pathway in cancer patients allows for targeted therapy to such specific pathways. The mechanism of CRC occurrence has been analyzed in detail. There are at least four carcinogenic pathways associated with colorectal oncogenesis, the ‘adenoma–carcinoma’ sequence type, ‘*de novo*’ type, HNPCC type and colitic cancer type.

## Adenoma–carcinoma sequence type

In 1988, Vogelstein *et al*, introduced a multi-step genetic model of colorectal carcinogenesis.[65] This model assumes that the *APC* gene mutation occurs at the first stage of the carcinogenesis process.[66] The *APC* gene has been identified as a causative gene of FAP and is involved in the regulation of β-catenin, cytoskeleton organization, apoptosis, cell cycle control and cell adhesion.[67] *APC* mutations occur in up to 80% of adenomas and adenocarcinomas and 4.3% of ACF.[68–70] APC protein translated from the *APC* gene is a main factor in the Wnt signal pathway and *APC* regulates cell proliferation by binding and degrading β-catenin protein that promotes cell proliferation.[71] However, the mutant APC protein cannot bind and degrade β-catenin protein and, as a result, β-catenin protein translocates to the nucleus and binds to the T-cell factor/lymphocyte enhancer factor transcription factor, which targets *c-myc*, *cyclin D1* and *c-jun* genes and promotes cell proliferation.[72,73] In fact, the *APC* ^Min^/ *cyclin D1*^−/−^ mice show reduced intestinal tumor number in animals genetically heterozygous or nullizygous for *cyclin D1.* [74] The K-*ras* gene is one of the oncogenes and it is assumed that the mutation occurs after the *APC* gene in the CRC.[70] Ras-guanosine 5'-triphosphate (GTP) binds cytoplasmic Raf- 1 and translocates it to the plasma membrane where Raf- 1 becomes activated by poorly understood mechanisms.[75,76] The signal is transmitted to the mitogen-activated protein kinase/extracellular signal-regulated kinase and extracellular signal-regulated kinase further downstream by this activation and promotes cell proliferation and differentiation.[77–79] Activating mutations in K-*ras* genes have been identified in a great variety of human carcinogenesis. The mutated forms are found to stimulate cell proliferation, transformation and differentiation.[80] *Ras* gene mutations occur in 58% of adenomas larger than 1 cm and in 47% of carcinomas. However, K-*ras* mutations are found in 9% of adenomas less than 1 cm in size.[65] In addition, due to the identification of the same point mutation in the same patient's adenoma and adenocarcinoma, it is thought that the mutation of the K-*ras* gene occurs during the early stage of carcinogenesis and is related in an increase of the size of the tumor. More than 90% of the primary CRCs with loss of LOH of chromosome 18q show a deletion in the colorectal carcinoma (*DCC*) gene included in the region of allelic loss.[70,81,82] Several studies have linked chromosome 18q LOH in CRCs to a reduction in DCC expression at the RNA level[82,83] and the protein level,[84–86] although some studies have failed to find evidence for reduced DCC transcript and/or protein levels in CRCs.[87] Therefore, the findings do little to establish whether *DCC* loss /inactivation is a critical factor in carcinogenesis or merely an epiphenomenon. However, recent studies reported that *DCC* functioned as part of a receptor complex for netrin-1.[88–90] Furthermore, in various cell lines, *DCC*, on ntrin-1 binding, activates the ERK pathway[91] and in the absence of netrin-1, induces apoptosis via caspase-9.[92–95] The presence of *netrin-1* blocks *DCC*-induced apoptosis.[94–96] A mutation of *DCC* that yields cell immortality is caused by continual transmission of the living signal in the absence of *netrin-1*. The most important point that determines the borderline between the adenoma and the adenocarcinoma is a mutation of the *p53* gene.[70] The *p53* gene is a typical tumor suppressor gene and its mutation has been detected in a variety of cancers, the mutation or LOH being present in about 75% of CRCs.[97] On the other hand, it is thought that this mutation is a conversion point from adenoma to adenocarcinoma because it is rarely detected in adenoma. Both intrinsic and extrinsic stress to the cell can act on the p53 pathway. The p53 protein acts as a cellular stress sensor and a rise in p53 levels causes arrest in the G_1_ phase of cell cycle, cellular senescence or apoptosis by inducing various target genes.[98–100] G_1_ arrest is part of a checkpoint response whereby cells with sustained DNA damage pause in G_1_ to allow for DNA repair before the cell cycle progresses. This mechanism limits the propagation of potentially oncogenic mutations. The p53-dependent apoptotic pathway is also induced by DNA damage in certain cell types as well as in cells undergoing inappropriate proliferation. The major players of the p53-induced cell cycle arrest are *p21*,[101] growth arrest and DNA damage inducible gene 45 (*GADD45*) genes.[102] The *p21* gene is widely accepted as a cyclin-dependent kinase inhibitor that can influence cell cycle progression from G_1_ to S phase by controlling the activity of CDK.[103] On the other hand, *GADD45* inhibits the cell progression from G0 to S phase and plays an important role in the maintenance of the stability of the chromosome.[104] Other major players of the *p53*-induced apoptosis are pro-apoptotic Bcl-2 protein Bax,[105] BH-3-only proteins Noxa[106] and *p53* upregulated modulator of apoptosis.[107] These proteins have the ability to induce mitochondrial *cytochrome c* release.[108,109] Thus, transactivation of their promoters through p53 might induce caspase activation. Therefore, it is thought that the loss of *p53* function as a transcription factor affects cellular malignant transformation.

## HNPCC-type pathway

The relationship between a stability gene aberration and CRC is revealed by HNPCC, also termed Lynch syndrome, research. This research started with a report by Warthin about the family G that experienced frequent colon, rectum, stomach and endometrium cancer in 1913.[110] Instability of short tandem repeats, or microsatellites (MSI), is a characteristic of the tumors from these patients.[111] In most HNPCC CRCs, MSI has been shown to result from mutations in the DNA mismatch repair, *hMSH2*, *hMLH1*, *hPMS1*, *hPMS2* and *hMSH6* genes.[112,113] Most microsatellite sequences in the genome are located within non-coding or intronic sequences and mutations in these introns are believed to be silent and inconsequential. However, genes may contain MSI within their coding regions. Some of these genes have been identified, including receptors for growth factors, such as transforming growth factor-β receptor II,[114] insulin-like growth factor-II receptor[115]), regulators for cell cycle[116] and regulators of apoptosis.[117] The transformation to malignancy thus occurs when these target genes are mutated.

## *de novo*-type pathway

Classical forms of CRC are characterized by the development from adenomatous polyps via the adenoma–carcinoma sequence, with progressive accumulation of genetic alterations. In the 1980s, several Japanese researchers began to report that they could detect flat-type carcinoma with a diameter of less than 10 mm arising *de novo*, which tended to reach the deeper layers at an earlier stage than polypoid-type carcinoma in adenoma.[118–120] This flat-type carcinoma[121] shows fewer mutations of the *APC* and K-*ras* genes than polypoid-type carcinoma, although mutations of the *p53* genes are seen at the same level as in polypoid-type carcinoma. However, the epigenetic inactivation of Ras-associated factor (*RASSF*) *1A* by hypermethylation of the promoter region is frequently detected in flat-type carcinoma. *RASSF1A* regulates a pro-apoptotic pathway through heterodimerization with the Ras effector *NORE1* and interacts with pro-apoptotic protein kinase *MST1*, which mediates the apoptotic effect of *Ras.*[122] Therefore, it is thought that the inactivation of *RASSF1A* causes an aberration in the ras-signaling pathway without involving the K-*ras* gene mutation. There is a significant inverse relationship between *RASSF1A* hypermethylation and K-*ras* mutations. These results suggest that the *RASSF1A* plays an important role with *p53* in the *de novo*-type of carcinogenesis pathway.

## Colitic cancer-type pathway

UC and CD are IBDs of unknown etiology. In 1925, Crohn and Rosenberg reported the first case of IBD-associated CRC. Since then, it was recognized that CRC occurred at a high rate in UC patients with extensive colitis of greater than 8–10 years duration.[123,124] In addition, flat dysplasia thought to be a pre-cancerous lesion occurs in colonic mucosa in IBD patients.[125] Hence, the IBD associated with CRC is believed to occur by a progression from no dysplasia to indefinite dysplasia to low-grade dysplasia to high-grade dysplasia to carcinoma. A novel mouse model of colitis-related colon carcinogenesis was recently established for investigating the pathogenesis of UC-related CRC development.[126] Several inflammation-related genes, transcribed by the common transcription factor, nuclear factor-κB (NF-κB),[127] such as cyclooxygenase (COX)-2,[128] inducible nitric oxide synthase (iNOS),[129] interferon-γ, tumor necrosis factor-α and interleukin-1β,[130] are increased in inflamed mucosa and remain elevated in colonic neoplasms. NF-κB is a central regulator of the transcriptional activation of a number of genes involved in cell adhesion, immune and pro-inflammatory responses, apoptosis, differentiation and growth. Induction of these genes in intestinal epithelial cells by activated NF-κB profoundly influences mucosal inflammation and repair. However, chronic activation of NF-κB induces promotion of epithelial cell turnover and generation of reactive oxygen and nitrogen species (RONs). It is thought that heightened epithelial cell turnover and DNA damage caused by RONs appear to drive the carcinogenesis processes. Several natural compounds suppress NF-κB expression and inhibit inflammation-related colon carcinogenesis.[131–134]

## Experimental colon carcinogenesis

The ability to reliably induce colon tumors in animals has provided the opportunity to study various aspects of the carcinogenesis process. These models have provided information on the initiation, promotion and progression of tumors, including detailed information on cellular transformation and the subsequent events leading to the formation of neoplastic lesions. The established models can be used for chemoprevention studies as well. These animal models are chemically induced[135–137] and genetically modified.[138,139] Oncogenesis studies using these models have also elucidated the role of genetic and environmental factors and other influences on the various aspects of this complex disease. Animal colon cancer models have also been used to evaluate immunological, chemical and surgical therapy regimens.

## Colonic carcinogens

There are chemical agents that have been used to induce colon tumors in animals. They include direct- and indirect-acting agents. The direct-acting carcinogens are compounds that do not require biological catalysis, such as the action of enzymes to form the ultimate reactive species that alters cellular macromolecules. These agents spontaneously break down in an aqueous environment to electrophilic species that react with nucleophilic centers on the DNA molecule. Indirect-acting carcinogens require enzymatic action to be converted into the electrophilic species. Many researchers deal mainly with experiments involving dimethylhydrazine and its metabolites AOM, DMH and methylazoxymethanol (MAM) acetate in the rodent colon tumor model. Most of the experiments involving chemical colon induction have been performed with these carcinogens using mice or rats. AOM is the most widely used colon carcinogen.

## Mechanisms of action

Initially, the carcinogens are converted to a positive charged molecule, customarily designated as an electrophile. The electrophile can then either be detoxified by reacting with a free electron pair in the cellular milieu or it can react with a negative center on DNA resulting in the formation of adducts on specific bases in the DNA. The chemically altered DNA can then be either restored by DNA repair enzymes or carry the errors through numerous rounds of duplication with the introduction of additional changes, finally leading to a cancerous cell. In addition to this sequence of events, the step between the altered DNA and the neoplastic event can often be enhanced by substances known as promoters. The initiation and promotion steps can also be decreased by agents known as inhibitors. A significant proportion of the studies using chemical carcinogens to initiate colon tumors have been designed to determine if various substances promote or inhibit the initiation step. The metabolism and mode of action of AOM and DMH were investigated by Fiala.[140] The initial step in the activation of a colon carcinogen such as AOM, DMH or 3,2'-dimethyl-4-aminobiphenyl (DMAB) is a series of oxidative steps that occur in the liver.[141] Oxidative steps are also involved in the activation of DMH. The compounds can then enter the intestine via the bile or the blood system. Generally, compounds, before entering the bile, are conjugated in the liver with glucuronic acid and/or sulfate or glutathione.[142] The conjugates entering the intestine can then be converted into free compounds by hydrolytic bacterial enzymes. The compounds formed in this sequence can then be activated to the ultimate electrophilic carcinogens by the action of colon tissue enzymes or, possibly, by the further action of bacteria plus colon tissue-activating systems. Bacteria are capable of deactivating proximal carcinogens, affording protection against tumor induction.[143] In this sequence, the initial activation occurs by the action of hepatic oxidative enzymes leading to the formation of MAM. It was initially thought that MAM spontaneously broke down to a methyl carbonium ion, which then alkylated the DNA. An alternate pathway exists,[144] in which the alcohol functional group on MAM is further oxidized by extrahepatic tissue dehydrogenases to the aldehyde and acid, which then spontaneously form the carbonium ion. It appears that both mechanisms occur simultaneously, making MAM both a direct and an indirect-acting carcinogen. In contrast to DMAB, the MAM molecule is small and therefore the transport from the liver to the intestine primarily occurs via the circulatory system.[140] However, a small percentage of MAM may be conjugated and excreted in the bile. Evidence for this also appears from the observation that animals receiving metabolites of DMH develop small intestinal tumors just distal to the point of entrance of the bile duct into the intestine.[145]

## Relevance of animal models to human CRC

**Figure 11:**
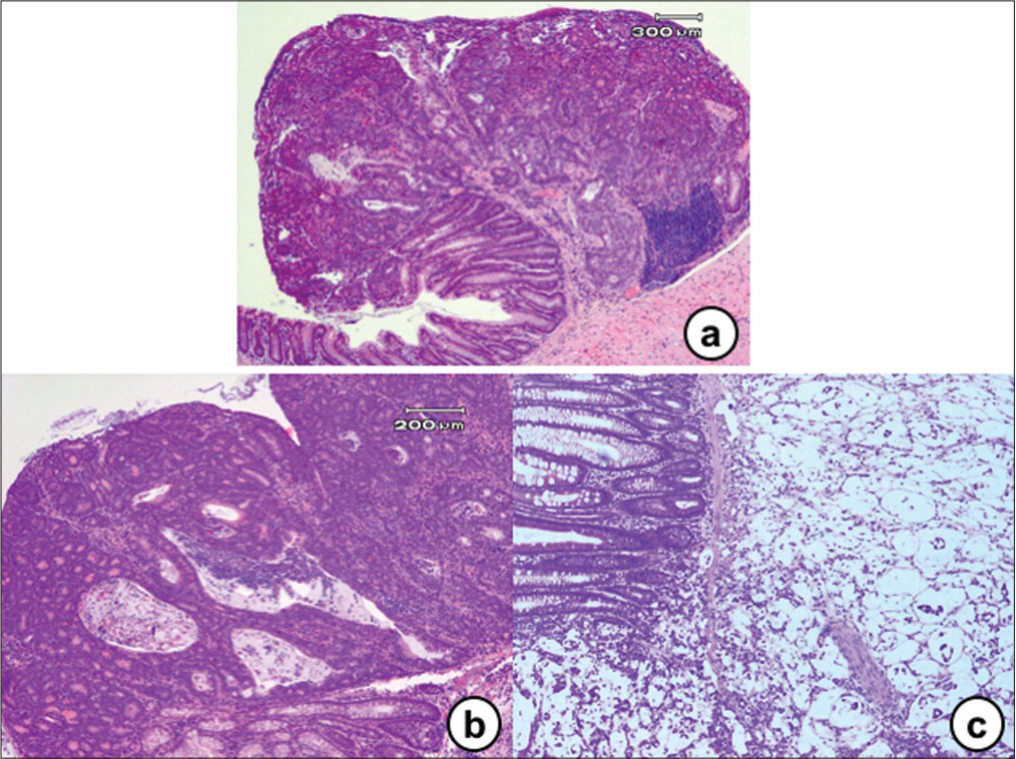
The histopathology findings of colonic adenocarcinomas induced by AOM in rats. (a) A polypoid tumor and (b) sessile adenocarcinomas. (c) A mucinous adenocarcinoma, Cancer cells are suspended in mucous lakes

There are many similarities between human colon cancer and AOM- or DMH-induced colon cancer in rats. There are also some characteristics that differ between the human and rodent diseases. The pathological features of DMH-induced colon tumors are the same as seen in the human disease. The animal tumors vary from tubular adenomas to polypoid and sessile carcinomas [Figure 11a and b] to mucinous adenocarcinomas [Figure 11c].[145] The kinetics of the colonic epithelial population show analogous alterations in the proliferative compartment in the human disease and the animal model.[146] There are also similarities in colonic mucin synthesis in human colonic neoplasia and AOM- or DMH-induced tumors.[147] There are, however, areas of discrepancy between human and chemically induced colon tumors.[135] There is a large body of evidence indicating that there is a transition from adenomatous polyps to CRC in humans. In the rat model of colon cancer, there are a number of studies that have shown no evidence of benign polyps before the development of colon cancer.[146,148] AOM- or DMH-induced rat colon adenocarcinomas arise *de novo* from flat mucosa and not from benign polyps.[146,148] However, adenomas develop in rodents.[149,150] This supports the progression from benign adenomas to polypoid adenocarcinomas and is consistent with an adenoma–carcinoma sequence similar to human colon carcinogenesis. There is another aspect, namely the metastatic potential of the colonic adenocarcinomas, which differ significantly between the animal model and the human disease.[135,151] In human CRC, approximately 50% of the patients have evidence of lymphatic metastases and 33% have hematogenous metastases at the time of diagnosis. In contrast, studies involving the AOM and DMH models of colon cancer show a very low incidence of metastases.[135] The metastases are generally limited to the regional lymphatics or the peritoneal surface. In the human disease, there is an orderly progression of the tumor through the lymphatic network. The usual route of hematogenous metastases is initially to the liver and, eventually, to the lung. Metastases to the liver are rare in rodents with AOM- and DMH-induced colon cancer. Although chemically induced colon tumors in animals have provided considerable information regarding the human disease, there are many research avenues that have not been fully utilized. More effort in establishing metastasis models and testing chemopreventive agents in animal models is required. There is a need for more experiments using colonoscopy[152] or other diagnostic tools to follow the pre-neoplastic events in the large bowel. The use of animal models to investigate carcinogens in food and the fecal stream should get more attention. The testing of compounds that may require a long series of activation steps and, therefore, are not obvious carcinogens, should also be encouraged. More effort is required in delineating the factors involved in the initiation and the early and late promotion steps of colon cancer. An increased research effort should be focused on single or multiple agents that can affect long-term changes in the intestinal environment, leading to a lower cancer incidence. The use of chemical agents to induce tumors in animals has been, and will continue to be, an important experimental model in the search to prevent and cure colon cancer.

## AOM/dexransodium sulfate (DSS) model for colitis-related colon carcinogenesis

Colon cancer is the most serious complication associated with longstanding IBD, including UC and Crohn's colitis.[153,154] The risk of CRC increases with the extent and duration of the disease. Several animal models have been reported that recapitulate many of the features associated with IBD. Perhaps the most commonly used mouse model employs DSS.[155] CRC development in the DSS colitis model typically requires a relatively long exposure period or cycles of DSS administration and the incidence and/or multiplicity of induced tumors is relatively low.[156]

**Figure 12:**
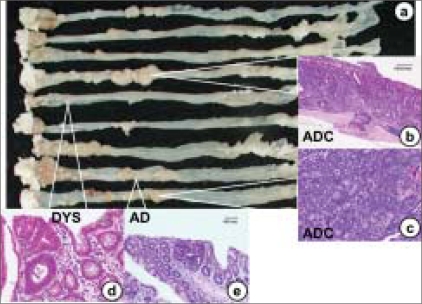
Colonic tumors developed in mice that received AOM and DSS. (a) Macroscopic view shows a number of colonic tumors in the distal colons. (b–e) Histopathology findings of colonic lesions induced by AOM and DSS. Some colonic adenocarcinomas invade the serosa (b). Most adenocarcinomas are of the well-differentiated type, but some moderately differentiated (b) and poorly differentiated types are also observed. Dysplastic crypts (d) are frequently observed in the mucosa, which develop many small adenomas (e)

Many studies have suggested that chronic or repeated mucosal inflammation may result in tumorigenesis through several proposed mechanisms. These mechanisms include induction of genetic mutations, increased cryptal cell proliferation, changes in crypt cell metabolism, changes in the bile acid enterohepatic circulation and alterations in the bacterial flora.[157,158] CRC development following DSS-induced inflammation supports the hypothesis that chronic inflammation in IBD plays a critical role in epithelial malignant neoplasia in the large bowel.[157] In inflammatory and/or angiogenic environments, superoxide anion may be produced by infiltrating mast cells[159] or macrophages[160] to facilitate cancer development. A novel colitis-related mouse CRC model (a two-stage mouse colon carcinogenesis model) initiated with AOM and promoted by DSS was developed to obtain a better understanding of the pathogenesis of IBD-related CRC.[126,135,161] In this model, mice initiated with a low dose of a colonic carcinogen, AOM (10 mg/kg body wt), develop tumors [Figure 12] after a relatively short-term DSS exposure. The subsequent dysplasia and neoplasms show positive staining for β-catenin, COX-2 and inducible nitric oxide synthase, but did not show evidence of p53 immunoreactivity. This novel mouse model combining AOM with DSS, thus, can be used for investigating colitis-related colon cancer. Interestingly, numerous mast cells are found surrounding or within these carcinomas.[161] Recent studies on inflammation-related carcinogenesis, where mice received a single low dose of various colon carcinogens (i.e., AOM, PhIP and DMH) followed by 1 week of exposure to 2% DSS, have shown a high incidence of tumor formation within 20 weeks.[162,163]

In addition, different degrees of sensitivity to AOM/DSS-induced colon carcinogenesis have been observed among the four different mouse lines (BALB/c, C3H/HeN, C57BL/6N and DBA/2N).[164] When these mice were given a single i.p. injection of AOM (10 mg/kg body wt), followed by 1% DSS (wt/vol, molecular weight 36,000–50,000) in drinking water for 4 days, the incidence and multiplicity of colonic adenocarcinoma of Balb/c mice was the highest, followed by C57BL/6N mice. Only a few colonic adenomas, but no adenocarcinomas, developed in C3H/HeN or DBA/2N mice. The inflammation and immunohistochemical nitrotyrosine positivity score of these mice treated with AOM and DSS was closely correlated with the occurrence of colonic tumors. These findings suggest that susceptibility to AOM/DSS-induced colonic tumorigenesis may be directly influenced by the degree of nitrosative stress in colonic tissue.

In a dose–response study of DSS as a tumor promoter,[165] male ICR mice were given a single i.p. injection of AOM (10 mg/kg body wt) followed by DSS (molecular weight 40,000) at dose levels ranging from 0.1 to 2% (wt/vol) in drinking water for 1 week. At week 14, the incidence and multiplicity of colonic tubular adenoma and adenocarcinoma were highest in the mice that received AOM and 2% DSS. Colon tumors did not develop in mice that received AOM/0.25% DSS and AOM/0.1% DSS, although dysplastic crypts were observed in these mice. Scoring of inflammation and nitrotyrosine immunoreactivity suggested that severe inflammation and nitrosative stress was caused by high doses (2 and 1%) of DSS. Therefore, the tumor-promoting effect of DSS is dose dependent, occurring at a concentration of 1% or higher, and the effect corresponds to the degree of inflammation and nitrosative stress, which were assessed in this study by the increased (two-fold) nitrotyrosine immunoreactivity that was observed within a variety of cell types (neoplastic, cryptal and endothelial cells as well as infiltrative mononuclear cells) within the colonic mucosa.[165] A time–course study on the sequential pathological alterations during AOM/DSS-induced carcinogenesis revealed that the AOM/DSS model generates neoplasms that develop through dysplastic lesions in the colonic mucosa.[166]

Although the incidence of colon tumors in *Apc^Min/+^* mice is generally low, numerous colon tumors developed within 5 weeks in male and female *Apc^Min/+^* mice when they received 2% DSS in drinking water for 7 days;[167] thus, suggesting the powerful tumor-promoting ability of DSS in the colon. Further molecular analysis revealed that mutations within codons 33 and 34 might be caused by AOM exposure, whereas codon 32 mutations result from DSS exposure.[163]

Gene expression was altered in the inflamed colon of mice that received a combination of AOM and DSS in comparison with that of untreated mice or mice treated with either AOM or DSS alone.[168] Significantly upregulated genes in the AOM/DSS group include *Wif1*, *Plat*, and *Myc* at week 5 and *Plscr2* at week 10. The combination of AOM/DSS also resulted in the downregulation of *Pparbp* at weeks 5 and 10 and *Tgfb3* at week 10. The impact of these changes in the gene expression on cancer development has not yet been evaluated.

Rats that receive AOM followed by DSS develop a number colonic tumors within a 20-week experimental period.[169] In summary, the AOM/DSS animal model with mice and rats has proven to be a powerful tool for investigating the pathogenesis and chemoprevention of colitis-related colon carcinogenesis. Modifications of this model might facilitate the detection of environmental carcinogens[170] and other tumor risk modifiers,[171] as well as novel chemopreventive agents[131,132,134,169,172] against IBD-related CRC.

## Chemoprevention

Chemoprevention,[173] a science that has emerged during the last decade, presents an alternative approach to reducing cancer-related mortality.[174,175] It involves the long-term use of a variety of oral agents that can delay, prevent or even reverse the development of adenoma in the colorectum and interfere with the multi-step progression from adenoma to carcinoma.[176] Chemoprevention is thus of particular importance to genetically predisposed patients and to those patients who are especially susceptible to the environmental causes of CRC.[177] Chemoprevention presents a plausible approach to reducing the incidence and mortality from cancer.[176,177] CRC is particularly suitable for prevention strategies because it is prevalent and associated with considerable morbidity and mortality.[178] CRC has a natural history of transition from normal crypts through adenoma (a benign epithelial neoplasm) to overt adenocarcinoma (a malignant epithelial neoplasm) occurring over an average of 10–20 years, thereby providing a window of opportunity for effective intervention and prevention. Large bowel carcinogenesis occurs via a multi-step process, characterized by molecular and cellular alterations.[43,179–182] Some of these changes include alterations in oncogenes, loss of tumor suppressor genes and abnormalities in genes involved in DNA repair,[59] These molecular alterations result in identifiable pre-neoplastic lesions, such as ACF, BCAC and adenomatous polyps. Interference with these processes forms the basis for chemoprevention, which attempts to target key molecular events along these pathways. Recent pre-clinical studies and clinical trials have provided data on the potential benefit of a number of nutritional or non-nutritional elements as well as drugs in the chemoprevention setting. In this report, only non-nutritional elements will be discussed for their supplementation in diets to inhibit colon carcinogenesis.

The most important molecular markers/targets involved in signal pathways are polyamine (ornithine decarboxylase), COX-2, iNOS, 3-hydroxy-3-methylglutatyl coenzyme A reductase, retinoid X receptor-α, estrogen receptor-β, β-catenin, 5-lipoxygenase, signal transducers and activators of transcription 3, NF-κB and hemeoxygenase-1. These can be also considered as biomarkers and/or surrogate endpoint markers in colorectal carcinogenesis and also enzymatic and molecular targets for colorectal malignancies.[181,182]

Reddy and Rao extensively studied potent chemopreventive agents against colon cancer development and clarified their mechanisms using a pre-clinical animal model.[183–189] Currently, genetic information about cancer, molecular signaling and metabolic pathways has been translated into specific therapy that targets specific molecules for prevention.[181,182] Developing new technologies to provide knowledge of the functions of non-coding RNA, such as small interfering RNA, microRNA and piwi interacting RNA, can form a basis for the development of novel chemopreventive agents[190] that intervene at different steps during the multi-stage carcinogenesis process: preventing the initial mutation, blocking promotion to pre-malignant tumors, stopping progression from the pre-malignant state to *in situ* carcinomas or preventing invasion or metastasis.[191] Because the early stages of tumor promotion and progression are rate limiting, successful targeting of molecular events during these stages can have a great impact on the outcomes.

## Conclusions and future perspectives

The basis of CRC has been determined from the clinical and pre-clinical studies. These revealed that no other malignancy shows such an abundance of pre-neoplastic and/or pre-cancerous lesions. Moreover, these lesions in human colon can be found and removed quite easily. The condition is far different from that of most malignant neoplasms affecting humans. CRC, thus, is preventable by interfering with the process of oncogenesis that begins with an uncontrolled growth in the initiated cryptal cells, continues with the formation of an adenomatous polyp or dysplasia and, eventually, after many years, evolves into epithelial malignancy. Even in this phase of oncogenesis, there is time for intervention because the localized lesions are curable with surgery in the large majority of cases and/or preventable by chemoprevention. Unfortunately, many people still die of CRC, which may be due to lack of relevant symptoms and to the reluctance of individuals to undergo appropriate screening. CRC might be prevented and defeated if the general population becomes aware of the importance of screening of the early lesions (adenomatous polyp and/or dysplasia) and of their removal. The use of pre-clinical animal models to investigate carcinogens in single or multiple agents that can affect long-term changes in the intestinal environment leading to lower cancer incidence should get more attention for prevention of CRC development in the general population. More effort is required in delineating the factors involved in the progression of colorectal oncogenesis and therefore increased research efforts should be focused on metastasis.
